# Discussion of Treatment Options for Metastatic Hormone Sensitive Prostate Cancer Patients

**DOI:** 10.3389/fonc.2020.587981

**Published:** 2020-10-15

**Authors:** Samantha S. Sigurdson, Francisco E. Vera-Badillo, Fabio Ynoe de Moraes

**Affiliations:** ^1^Division of Radiation Oncology, Department of Oncology, Kingston Health Sciences Centre, Queen’s University, Kingston, ON, Canada; ^2^Division of Medical Oncology and Hematology, Department of Oncology, Kingston Health Sciences Centre, Queen’s University, Kingston, ON, Canada

**Keywords:** systemic therapy, hormone therapy, radiotherapy, metastatic prostate cancer (mPCa), cost-effective

## Background

The revolution in the management of patients with metastatic hormone sensitive prostate cancer (mHSPC) began in 2015 with the addition of novel systemic therapies to androgen deprivation therapy (ADT). Prior to this single agent ADT with a luteinizing hormone-releasing hormone agonist or receptor antagonist was the standard of care. Due to advances in biology and imaging there is now better understanding that halting the molecular evolution of the primary tumor can delay the development of local symptoms and initiation of the metastatic cascade or progression of existing metastases (1). Recommendations for systemic therapy are based on randomized control trial (RCT) evidence with positive overall survival (OS) benefit (docetaxel, abiraterone, enzalutamide, and apalutamide) (2–7). Patients can be categorized as either high (two of the following three factors: Gleason ≥8, ≥3 bone lesions, or visceral metastasis) (8) or low risk metastatic (M1) disease; or high (HVD; visceral metastases and/or ≥4 bone metastases with at least 1 outside vertebral column and pelvis) (9) or low volume disease (LVD) patients. One RCT found improved OS in a subgroup analysis of LVD patients treated with local radiotherapy (RT) (10). We review recent RCTs investigating approaches to managing mHSPC since new clinical and biological evidence clearly indicate a clinical conundrum when defining best first line treatment.

Ten trials (summarized in **Supplementary Table 1**) assessed experimental interventions in mHSPC patients in addition to ADT. These trials have comparable populations and there were no major differences between patients enrolled in the systemic and radiation therapy trials, except the non-RT STAMPEDE trials included high risk localized disease or node positive patients, though the majority of patients in both trials presented with mHPSC (3, 4). The age and performance status (PS) of the patients were comparable as 64-93% had a PS of 0 and the median age range was 63-68 years old. The proportion of patients with HVD ranged from 50-80%.

## Systemic Therapy

Docetaxel plus ADT has been studied in three RCTs with OS as the primary endpoint, first the negative GETUG-15 trial and subsequently the positive CHAARTED (HR = 0.72) and STAMPEDE Dox (HR=0.81 in M1 patients) trials, that found an OS benefit of 10 months compared to ADT alone (2, 3, 11, 12). In the CHAARTED trial the subgroup of HVD patients had a greater OS benefit of 17 months from the addition of docetaxel (9). There was no significant difference in the LVD group, which was confirmed after a median follow up of 54 months (9). In GETUG-15, there was a non-significant additional 5 month OS benefit in the HVD subgroup (11). However, after 78 months of follow up the STAMPEDE Dox trial did not find evidence that the benefit of docetaxel differed by metastatic burden (12). This difference between the trials may be due to the varying study populations, as about 95% of STAMPEDE Dox patients were *de-novo* presentations of metastatic disease and thus irrespective of their metastatic burden patients tended to benefit from the addition of docetaxel (12), while only about 75% were *de-novo* presentations in GETUG-15 and CHAARTED (9, 11). There was a significantly higher risk of adverse events (AEs), especially febrile neutropenia, with the addition of docetaxel in both the STAMPEDE Dox and CHAARTED trials,(2, 3). In the STAMPEDE Dox trial 52% of patients in the docetaxel + ADT arm experienced grade 3-5 AEs, compared to 17% in the ADT alone arm (3). Importantly, the GETUG-15 trial reported 4 docetaxel related deaths, two of which were neutropenia related (11).

Abiraterone was studied in two RCTs, STAMPEDE Abi and LATITUDE (4, 5). Abiraterone in addition to ADT was effective in all subgroups in the STAMPEDE Abi trial with a 7% improvement in 3-year OS (HR 0.63) in the abiraterone + ADT arm compared to ADT alone (13), though the effect was more pronounced for mHSPC patients (HR 0.61 vs. 0.75) (4). The STAMPEDE Abi trial did not report median OS in months and only half of patients had metastatic disease (4). The LATITUDE trial demonstrated the addition of abiraterone had a 16 month (HR 0.66; p<0.0001) improved OS (5). Though LATITUDE demonstrated the HVD subgroup had a significant OS benefit with a similar HR (0.62) compared to the primary analysis (5), the subgroup analysis by metastatic burden was underpowered, as well as questionable since the LATITUDE trial only enrolled high risk patients (13). Thus, some patients with LVD (<4 bone metastases) were excluded (13). Both trials reported abiraterone was overall well tolerated, though the STAMPEDE Abi intervention arm had a 47% rate of grade 3-5 AEs (9 deaths), compared to 33% and 3 deaths in the ADT arm (4, 5).

Enzalutamide has been studied in two large RCTs, ENZAMET and ARCHES, in mHSPC patients who could also be treated with ADT and docetaxel (6, 14). In the ARCHES trial there were not enough deaths to analyze the OS data (14), however in ENZAMET the 3 year OS was significantly improved by 8% (HR 0.67) (6). There was a higher frequency of serious AEs in the intervention arm of the ENZAMET trial, likely due to the longer duration of treatment as the events per person-year of exposure was proportional between the two arms (6).

One large RCT (TITAN) compared apalutamide to standard of care in over 1000 mHSPC patients (63% with HVD) (7). There was an 8% (HR=0.67) significant improvement in 2 year OS and the use of apalutamide was favorable for both LVD and HVD groups (7). There was no difference in toxicity as just over 40% experienced grade 3-4 AEs in both arms (7).

In summary, all four systemic agents now play a role in the initial management of mHSPC patients. Docetaxel or abiraterone should be considered for younger HVD patients with good PS who are prepared to accept the risks of toxicity. Docetaxel is given for 4 months but is associated with a significant (19-52%) risk of serious AEs (grade 3 or higher), including febrile neutropenia, while abiraterone is a continuous oral agent with a 23-47% risk of serious AEs (grade 3 or higher), including cardiac and hepatic toxicity (2–5). Enzalutamide and apalutamide are additional systemic agents with OS benefit in combination with ADT, though serious adverse effects rates are around 24% and 42% for enzalutamide and apalutamide respectively. Finally, when docetaxel is combined with enzalutamide there is increased frequency of severe AEs, there were 184 grades 3-4 AEs in 159 patients who received both drugs, and 19 in 404 patients who received enzalutamide and not docetaxel (6). In the enzalutamide arm without docetaxel there were <1% grades 3-4 AEs of any nervous system disorders or hematologic AEs, while the rate was 3% and 22% respectively for those who received both drugs (6).

## Radiation Therapy

Two negative RCTs, STAMPEDE RT and HORRAD, investigated the OS benefit of localized RT to the prostate. There were interesting findings from the STAMPEDE RT pre-planned subgroup analyses of LVD patients as there was a median OS benefit of 4 months (HR 0.68; p = 0.007) in the RT + standard of care (SOC) arm compared to SOC alone, and this trend was repeated with the same, though non-significant, HR (0.68) in the HORRAD trial between the RT + ADT and ADT alone arms (10, 15). The HORRAD trial may have been negative due to its protocol being developed in the 2000s, when the prescribed dose in standard fractionation was 70 Gy, lower than the now standard of care escalated dose of up to 80 Gy (15). Additionally, only bone scintigraphy was required for staging, and the now more commonly used computed- or prostate-specific membrane antigen positron-emission-tomography scans likely lead to metastatic disease being detected earlier, thus patients who were previously classified and treated as M0 could now be M1 (15). For this reason the HORRAD authors were unable to ascertain the presence of visceral metastases or pelvic lymphadenopathy, and thus disease burden was analyzed by high (≥5 bone metastases) or low disease burden, while STAMPEDE RT used the well known HVD and LVD classifications (15). This may have also affected the analysis results (15). RT is well tolerated with only 5% grades 3 to 4 AEs reported in the STAMPEDE RT intervention arm (10). Since this therapy has low risk of serious AEs and can improve OS, local RT should be considered for patients with LVD and can be offered to patients when the use of systemic agents could be unsafe, for example, 70 years and older and/or lower PS, due to the higher risk of grade 3-4 toxicity.

## Discussion

As trials in the mHSPC population continue to progress we predict that the optimal treatment modality will vary by pre-specified groups, patient characteristics, and cost. Updated 2020 guidelines from the European Association of Urology (EUA), European Society for Medical Oncology (ESMO), and National Comprehensive Cancer Network (NCCN) indicate docetaxel, abiraterone, enzalutamide, apalutamide, or localized RT to the prostate are treatment options for mHSPC (16–18). The surge in new drugs available to treat metastatic prostate cancer has led to concerns about costs, especially in low- and middle-income countries. An economic analysis found ADT had a total lifetime cost of $205,573 (USD), while docetaxel with ADT was $216,057, and abiraterone plus ADT was much more expensive at $669,177 in the mHSPC population (19). The incremental cost-effectiveness ratio (ICER) indicated treatment with docetaxel and ADT represented the most high value treatment, and abiraterone would have to be less than $3114 per month to be cost effective (19). A recently published cost-effectiveness comparison of 3 drugs (apalutamide, enzalutamide, and darolutamide) combined with ADT, and ADT alone, in metastatic castrate resistant prostate cancer (mCRPC) patients found the lifetime cost of ADT was $187,264, and in decreasing cost the combination chemohormonal therapy was apalutamide ($512,620), enzalutamide ($458,640), and darolutamide ($379,932) (20). These tended to exceed commonly accepted willingness-to-pay thresholds (<$100,000/quality-adjusted life-year (QALY)), though apalutamide combined with ADT had the lowest ICER and was most likely to be cost-effective on the basis of greatest QALY gains despite higher costs (20).

In the global oncology community there have been several regional panels to determine mHSPC management strategies taking into consideration local resources and the 2017 Advanced Prostate Cancer Consensus Conference (APCCC) recommendations (21). In the Middle East, a 2017 panel indicated the treatment of choice for mHSPC patients with either LVD or HVD is 6 cycles of docetaxel since it is more cost-effective than abiraterone plus prednisone until progression, even when generic forms of abiraterone become available (22). In Malaysia, a middle-income country, a 2018 panel of oncologists agreed with the APCCC to only add docetaxel for HVD patients, not in LVD (23). The panel noted in the ideal world abiraterone and prednisolone should be added to ADT for patients presenting with HVD, however in the real world setting the majority indicated treatment would be either chemohormonal therapy or ADT (23). The Asian Pacific regional group indicated cost and access issues are major influences in the real-world application of best practices (24). An economic analysis of men with mCRPC prescribed enzalutatimde compared to abiraterone found the enzalutamide group had lower all-cause outpatient costs due to lower number of visits, and lower pharmacy costs (25). Thus, in resource stricken countries ADT is the most cost effective therapy for mHSPC patients, and if chemohormonal therapy can be combined with ADT then 6 cycles of docetaxel is likely most cost effective, though additional economic analyses are required in the mHSPC population to investigate the cost effectiveness of the new chemohormonal agents available to this patient population.

In summary, docetaxel or abiraterone combined with ADT should be offered to mHSPC patients, especially good PS patients with HVD, who understand the potential associated high risk of grades 3-4 AEs. Enzalutamide or apalutamide are safe options for mHSPC patients across disease volume subtypes (6, 7). Until head to head trial data are available, clinical gestalt, cost, and patient preferences should be taken into consideration when deciding which systemic agent to combine with ADT. Finally, for mHSPC patients presenting with LVD, local RT combined with ADT should be considered due to reported OS improvement, minimal toxicity, and potential immunomodulation effect (10).

Take home message: The optimal treatment modality in the mHSPC population varies by disease burden and patient characteristics, and requires shared decision making between clinicians and patients. We believe future data will find two diverse mHSPC groups based on disease burden with differing treatments and prognoses.

## Author Contributions

All authors contributed to the article and approved the submitted version.

## Conflict of Interest

The authors declare that the research was conducted in the absence of any commercial or financial relationships that could be construed as a potential conflict of interest.
