# Supplementary material for: Discussion of Treatment Options for Metastatic Hormone Sensitive Prostate Cancer Patients
Source: Front Oncol. 2020 Oct 15;10:587981. doi: 10.3389/fonc.2020.587981 (PMC7594623; doi:10.3389/fonc.2020.587981)
Supplement: Supplementary file 1 [file Table_1.docx]

**Table 1 (online only): Summary of phase III RCTs investigating treatment options for metastatic castrate sensitive prostate cancer patients.**

| **Trial** | **Population** | **Intervention** | **Outcome** | **Toxicity** |
| --- | --- | --- | --- | --- |
| GETUG 15-  Dox + ADT vs. ADT^9^ | - 385 mHSPC patients - 71% *de-novo* metastatic presentation - Median age: 64 years old - HVD: 48% - ECOG PS 0: 93% | Dox 75 mg/m^2^ IV every 3 weeks for up to 9 cycles | - Median f/u: 50 mo. in initial paper; subsequent analysis after 84 mo. - Median OS 62 mo. (Dox+ADT) vs. 49 mo. (HR 0.88; p=0.3) - HVD subgroup ns difference of 5 mo.; 40 (Dox+ADT) vs. 35 mo. (HR 0.78; p=0.14) - LVD median OS not reached: ns | 19% serious AEs in Dox+ADT; 21% neutropenia, 3% febrile neutropenia, abnormal liver function tests 2%. 4 treatment related deaths, 2 of which neutropenia related, no deaths after granulocyte colony-stimulating factor initiated. No serious AEs in ADT group. |
| CHAARTED- Dox + ADT vs. ADT^2,11^ | - 790 mHSPC patients - 73% *de-novo* metastatic presentation - Median age: 63 years old - HVD: 65% - ECOG PS 0: 70% | Dox 75 mg/m^2^ every 3 weeks for up to 6 cycles | - Median f/u: 54 mo. - Median OS: 58 (Dox+ADT) vs. 47 mo. (HR 0.72; p=.0018) - HVD (n=513) OS 51 (Dox+ADT) vs. 34 mo. (HR 0.63; p<.001) - LVD or prior local therapy: ns. - HVD and *de-novo* presentation with mHSPC (n=421): OS 48 (Dox+ADT) vs. 33 mo. (HR 0.63, p<.001) | Grade 3-4 AE (Dox+ADT) 29.6%: febrile neutropenia 6.1% and with infection 2.3%.  QoL: FACT-P statistically worse for Dox+ADT group at 3 mo. compared to ADT group (p=.02) but significantly better at 12 mo (p=.04). ns clinical difference. |
| STAMPEDE Dox – Dox + ADT vs. ADT  (excluding zoledronic acid arm from this review^3,10^ | - 2962 patients, 61% mHSPC (otherwise high risk localized or N+ disease) - Median age: 65 years old - ECOG PS 0: 72% | Dox 6 cycles + prednisone 10 mg daily (n=592 Dox+ADT) | - Median f/u: 78 mo. - Median OS for M1 patients: 59 (Dox+ADT) vs. 43 mo. (HR 0.81; p=.009) - All other subgroup analyses ns, specifically no heterogeneity of effect stratified by metastatic burden | Grade 3-5 AE 52% (Dox+ADT) vs. 17%; 15% vs. 1% febrile neutropenia.  One grade 5 treatment related event in Dox+ADT arm (neutropenic sepsis). No evidence of worse late toxicity between the arms. |
| STAMPEDE Abi- Abi + ADT vs. ADT^4^ | - 1917 patients, 52% mHSPC - LVD and low risk metastatic disease: 48% of mHSPC subset - Median age: 67 years old - ECOG PS 0: 78% | - Abi 1000 mg daily + prednisone 5 mg daily - Median duration 23.7 mo. | - Median f/u: 40 mo. - 3 yr. OS 83% (Abi+ADT) vs. 76% (HR 0.63; p<.001) - Subgroup analysis by metastatic status demonstrated no heterogeneity of effect (p=0.37); however, M1 subgroup had significant HR 0.61 (95% CI 0.49-0.75) while M0 ns | Rate of grade 3-5 AE 47% (Abi+ADT, 9 grade 5 events) vs. 33% (ADT, 3 grade 5 events); 5% vs. 1% hypertension; 7% vs. 1% hepatic disorders. |
| LATITUDE- Abi + ADT vs. ADT^5^ | - 1199 *de-novo* mHSPC patients (all high risk metastatic) - LVD: 20% - Median age: 67 years old - ECOG PS 0 or 1: 97% | - Abi 1000 mg daily + prednisone 5 mg daily - Median duration 26 mo. | - Median f/u: 52 mo. (planned intermittent analysis led to unblinding and crossover allowed after median f/u 30 mo.) - Median OS: 53 (Abi+ADT) vs. 37 mo. (HR 0.66; p<0.0001) corresponding to a 3 yr. OS: 66% (Abi+ADT) vs. 49% - HVD subgroup: 50 (Abi+ADT) vs. 33 mo. (HR 0.62; p<0.0001) - LVD subgroup: ns (not adequately powered) | Grade 3-5 AE 32% (Abi+ADT) vs. 25% and 6% in crossover group to Abi+ADT; 23% vs. 11% vs. 4% hypertension; 16% vs. 2% vs. 3% hypokalemia.  3 treatment related deaths in each arm.  Improved QoL in Abi+ADT arm (PRO). |
| ENZAMET- Enza vs. SOC^6^ | - 1125 mHSPC patients; 52% HVD - 61% of HVD and 27% of LVD patients received Dox - Median age: 69 years old - ECOG PS 0: 72% | - Enza 160 mg daily - At 3 years 62% of Enza arm receiving a trial regimen | - Median f/u: 34 mo.; after first interim analysis RCT unblinded - 3 yr. OS: 80% (Enza) vs. 72% (HR 0.67; p=0.002); median OS not reached - 3 yr. PFS: 67% (Enza) vs. 37% (HR 0.39 p<0.001) - Subgroup analyses: trend improved OS in LVD (HR 0.43; p=0.14) and no early Dox planned (HR 0.53; p=0.14) | Frequency of serious AEs similar between two groups per person-year of exposure to a trial regimen; seizures 7 patients (1%; Enza) vs. 0% (SOC); fatigue ≥ grade 2 25% (Enza) vs. 14%. Higher rate AEs in patients receiving Dox; ≥ grade 2 peripheral neuropathy 11% (Enza) vs. 3%. |
| ARCHES- Enza vs. SOC^13^ | - 1150 mHSPC patients - 63% HVD - Median age: 70 years old - ECOG PS 0: 78% | - Enza 160 mg daily - Median duration 13 mo. | - Median f/u: 14 mo. - Risk of rPFS or death median not reached (Enza) vs. 19 mo. (HR 0.39; p<0.001) - Subgroup analyses: ns by volume of disease | Grade 3 or worse AEs 24% (Enza) vs. 26% |
| TITAN- Apa + ADT vs. ADT^7^ | - 1052 mHSPC patients - 16% received prior localized RT - 11% had received Dox - HVD: 63% - Median age: 69 years old - ECOG PS 0: 64% | - Apa 240 mg daily | - Median f/u: 23 mo. - 2 yr. OS 82% (Apa+ADT) vs. 74% (HR 0.67; p=0.005) - Subgroup analyses by volume of disease both demonstrated significant effect: LVD HR 0.36 (95% CI 0.22-0.57); HVD HR 0.53 (95% CI 0.41-0.67) - Significant OS benefit for subgroup not previously treated with Dox HR 0.63 (95% CI 0.47-0.85); subgroup treated with Dox ns | Grade 3-4 AEs 42% (Apa+ADT) vs. 41%; rash more common in Apa+ADT arm. |
| STAMPEDE RT- RT + SOC vs. SOC (18% Dox)^8^ | - 2061 mHSPC patients - LVD: 40% - Median age: 68 years old - WHO PS 0: 71% | Either:   - 36 Gy in 6 weekly fractions - 55 Gy in 20 daily fractions over 4 weeks | - Median f/u: 37 mo. - Median OS: 43 (RT) vs. 42 mo. ns (HR 0.92; p=0.266) - Prespecified subgroup analysis LVD reached significance: 49 vs. 45 mo. (HR 0.68; p=0.007) | In RT arm grade 3-4 toxicity was 5% acute and 4% late |
| HORRAD-  RT + ADT vs. ADT^14^ | - 432 mHSPC patients - Low disease burden: 37% <5 bone mets - Median age: 67 years old - WHO PS 0: 84% | Either:   - 70 Gy in 35 daily fractions over 7 weeks - 57.76 Gy in 19 fractions three times a week over 6 weeks | - Median f/u: 47 mo. - Median OS: 45 (RT+ADT) vs. 43 mo. (ns, p=0.4) - Trend improved survival in <5 mets subgroup (HR 0.68; 95% CI 0.42-1.10) | Not yet available. |

Dox: Docetaxel; ECOG PS: Eastern Cooperative Oncology Group Performance Status; f/u: follow up; mo.; months; OS: overall survival; HR: hazard ratio; ns: not significant; AE: adverse events; QoL: quality of life; FACT-P: Functional Assessment of Cancer Therapy-Prostate, N+: node positive; Abi: Abiraterone acetate; PRO: patient reported outcomes; Enza: Enzalutamide; SOC: standard of care includes ADT and docetaxel at discretion of treating physician; rPFS: radiographic progression free survival; Apa: Apalutamide; WHO PS: World Health Organization Performance Score; mets: metastases
